# Seasonal Changes Affect Root Prunasin Concentration in *Prunus serotina* and Override Species Interactions between *P. serotina* and *Quercus petraea*

**DOI:** 10.1007/s10886-016-0678-y

**Published:** 2016-03-09

**Authors:** Piotr Robakowski, Ernest Bielinis, Jerzy Stachowiak, Iwona Mejza, Bartosz Bułaj

**Affiliations:** Department of Forestry, Poznan University of Life Sciences, Wojska Polskiego 71E St., 60-625 Poznan, Poland; Department of Chemistry, Poznan University of Life Sciences, Wojska Polskiego 75 St., 60-625 Poznan, Poland; Department of Mathematical and Statistical Methods, Poznan University of Life Sciences, Wojska Polskiego 28 St., 60-637 Poznan, Poland

**Keywords:** Biomass allocation, Black cherry, Cyanogenic glycosides, Invasive species, Liquid chromatography, Oak, Photosynthesis, Prunasin, *Prunus serotina*

## Abstract

The allocation of resources to chemical defense can decrease plant growth and photosynthesis. Prunasin is a cyanogenic glycoside known for its role in defense against herbivores and other plants. In the present study, fluctuations of prunasin concentrations in roots of *Prunus serotina* seedlings were hypothesized to be: (1) dependent on light, air temperature, and humidity; (2) affected by competition between *Prunus serotina* and *Quercus petraea* seedlings, with mulching with *Prunus serotina* leaves; (3) connected with optimal allocation of resources. For the first time, we determined prunasin concentration in roots on several occasions during the vegetative season. The results indicate that seasonal changes have more pronounced effects on prunasin concentration than light regime and interspecific competition. *Prunus serotina* invested more nitrogen in the synthesis of prunasin under highly restricted light conditions than in higher light environments. In full sun, prunasin in roots of *Prunus serotina* growing in a monoculture was correlated with growth and photosynthesis, whereas these relationships were not found when interspecific competition with mulching was a factor. The study demonstrates that prunasin concentration in *Prunus serotina* roots is the result of species-specific adaptation, light and temperature conditions, ontogenetic shift, and, to a lesser extent, interspecific plant-plant interactions.

**Electronic supplementary material:**

The online version of this article (doi:10.1007/s10886-016-0678-y) contains supplementary material, which is available to authorized users.

## Introduction

Prunasin [(*R*)-mandelonitrile-ß-D-glucopyranoside] is a cyanogenic glycoside, a class of specialized plant metabolites. It is present in more than 3000 plant species, including economically important species such as sorghum (*Sorghum bicolor* L. (Moench)) and cassava (*Manihot esculenta* Crantz.) (Swain and Poulton 1994a). Prunasin occurs in plant tissues of *Rosaceae*, *Polypodiaceae*, *Myrtaceae*, *Saxifragaceae*, *Scrophulariaceae*, and *Myoporaceae*, but is especially abundant in *Rosaceae* and *Polypodiaceae*, where it is found in all plant organs (Vetter 2000).

In the present study, prunasin was determined for the first time in roots of *Prunus serotina* seedlings under different light and temperature conditions on several occasions during the vegetative period. So far, only a few studies have considered prunasin concentration in roots, and one study found that bitter-kernelled genotypes had higher prunasin levels in their roots than non-bitter genotypes (Dicenta et al. 2002). The other cyanogenic glycosides, linamarin and lotaustralin, have been studied in cassava roots (Hernández et al. 1995; Okafor 2005). Linamarin levels in cassava roots depended on cultivar, and were around 20 times lower compared to linamarin levels in leaves (Okafor 2005).

Several studies have shown significant differences in prunasin concentrations in leaves and twigs among species, varieties, cultivars, and individuals belonging to the same population (Burns et al. 2012; Goodger et al. 2004). For example, average prunasin concentrations in newly initiated leaves of *Amelanchier alnifolia* var. *cusickii* were three times greater than in *A*. *alnifolia* var. *alnifolia* (Brooke et al. 1984). Variations in the amount of cyanogenic glycosides can be attributed to differences in rates of their synthesis and to enzymatic degradation (Gleadow and Møller 2014; Vetter 2000).

In the case of tissue disruption, prunasin is degraded to hydrogen cyanide, glucose, and benzaldehyde by β-glucosidase prunasin hydrolase and mandelonitirile lyase (Sánchez-Pérez et al. 2012). After being hydrolyzed by endogenous enzymes, prunasin decomposes into hydrogen cyanide (HCN), a respiratory poison that inhibits the activity of metalloenzymes, including cytochrome *c* oxidase (Leavesley et al. 2008). Highly toxic for aerobic organisms, HCN inhibits respiratory function in cells, and thus impedes seed germination and plant growth rates (Gleadow and Møller 2014). HCN is produced when plant tissue has been damaged, which suggests that prunasin may defend cyanogenic plants against herbivores (Agrawal et al. 2012; Ballhorn et al. 2011a; Swain and Poulton 1994b). However, cyanogenic compounds do not increase resistance to pathogens (e.g., Lieberei 2006), but plants containing cyanide glycosides are less attractive to insects. For example, when feeding intensities of adult Japanese beetles [*Popillia japonica* (Newm.)] were compared among 27 taxa of *Prunus* (including *Prunus serotina*), feeding decreased exponentially as endogenous foliar cyanide production increased (Patton et al. 1997). The results of numerous other studies confirm that cyanogenic glycosides, including prunasin in roots (Dicenta et al. 2002; Mulas 1994), play a crucial role in plant defense against pests. Additionally, prunasin functions as a repellent and an N-storage compound, similar to other cyanogenic glycosides such as linustatin (Selmar et al. 1988; Swain and Poulton 1994b). The potential pathways for prunasin metabolism as well as HCN reassimilation in *P*. *serotina* seedlings were proposed by Swain and Poulton (1994b).

Usually, developing leaves show high levels of HCN, and HCN levels decrease with leaf age (Brooke et al. 1984; Majak et al. 1980). In *Eucalyptus cladocalyx* F. Muell., HCN production gradually decreased during the vegetative season, when photosynthetic capacity and growth rate are increased (Gleadow et al. 1998; Gleadow and Woodrow 2000). Cyanogenic capacity in leaves of *Eucalypthus polyanthemos* is influenced largely by genetic factors, while environmental aspects, such as soil nitrogen content, have a smaller influence (Ballhorn et al. 2011a; Goodger et al. 2004). However, much less is known about variations of prunasin concentrations in roots of cyanogenic woody plants and how these concentrations depend on ecological factors and ecophysiological regulatory mechanisms.

Du et al. (1995) showed that cynanogenic glycosides in cassava are not only translocated from leaves to roots, but also synthesized in roots. However, there is a lack of information about the possible light effect on regulation of the synthesis and translocation of the cyanogenic glycosides. Miller et al. (2004) showed that there were no differences in cyanogenic glycoside concentrations in foliage, stem, or roots of *Prunus turneriana* (F.M. Bailey), Kalkman under different light treatments. When the effect of light on cyanogenic glycoside content was studied, conflicting results were obtained (Gleadow and Møller 2014). In leaves of *Eucalyptus cladocalyx* F. Muell., cyanogenic glycosides were decreased by shading (Burns et al. 2002). In contrast, *Pteridium aquilinum* and *Trifolium repens* showed higher concentrations of cyanogenic compounds in shade compared with plants adapted to full light (Cooper-Driver et al. 1977; Vickery et al. 1987). Our study is the first to investigate the effect of light acclimation of *P*. *serotina* seedlings on prunasin concentrations in roots.

Plant species growing within a community interact both above- and belowground (McHugh and Gehring 2006). In the case of *P. serotina*, the belowground plant-plant interaction may be affected by cyanogenic exudates released from roots that affect roots of other plants (Badri and Vivanco 2009; Bais et al. 2004, 2006). Such allelopathic interactions between cyanogenic plant species and their competitors may contribute to the biodiversity of plant communities (Del-Val and Crawley 2005). However, transport of cyanide in soils is mostly influenced by volatilization and decomposition. Accordingly, the high volatility of cyanide and soil microbial activity ensure that high levels of cyanide do not persist or accumulate in soil under natural conditions (Ubalua 2010). On the other hand, cyanide derived from cyanogenic glycosides, including prunasin, may form stable complexes with metals (Fe^2+^, Mn^2+^, and Cu^2+^), making essential metals unavailable for plants (Ubalua 2010).

Genetic, physiological, and environmental effects on cyanogenic glycoside variability are poorly understood, particularly in tree species (Goodger et al. 2004). Approaches to define the growth environment and interspecific interactions are necessary to gain a better understanding of the role of prunasin in roots. Correlations between growth, physiological traits, and prunasin concentration in *P*. *serotina* roots growing in competition with *Q*. *petraea* under different light regimes should add insight into the life strategy of an invasive, cyanogenic species. According to optimal resource allocation theory, it has been assumed that an increase in prunasin concentration in *P*. *serotina* roots would be at the costs of a decrease in photosynthesis and growth (McKey 1974; Miller et al. 2004). A trade-off can be established between resource allocation into defense and growth (Gleadow et al. 2014). To our knowledge, no other studies have examined prunasin concentration in *P*. *serotina* roots growing in competition with another tree species under different light regimes.

The objective of this study was to investigate changes in prunasin concentration in *P*. *serotina* roots in response to climatic conditions and competition with *Q*. *petraea* seedlings. We posited that prunasin in *P*. *serotina* roots would fluctuate during the vegetative season, depending on sampling time and climatic parameters. In some cases, shade plants are more vulnerable to herbivory than sun plants (Karolewski et al. 2010), and thus, prunasin concentrations might be higher in seedlings acclimated to shade compared to those growing in full sun. Interspecific competition, along with mulching with *P*. *serotina* leaves, also could alter prunasin concentrations in *P*. *serotina* roots. We made an attempt to elucidate relationships between prunasin concentration and morphological and physiological traits of *P*. *serotina* seedlings within the hypothesis of optimal resource allocation.

## Methods and Materials

### Plants

*Prunus serotina* (Ehrh.) Borkh. is a deciduous tree or a small understory shrub native to North America. It grows under a wide range of climatic conditions. In its natural range, average annual precipitation is 970 to 1120 mm, and mean annual temperatures are below 24 °C. In the center of the natural range, January temperatures reach a maximum of 1 to 6 °C and a minimum of −11 to −6 °C. July temperatures reach a maximum of 27 to 29 °C and a minimum of 11 to 16 °C. *Prunus serotina* grows on a variety of soils, but does not tolerate very wet or dry conditions (Marquis 1990). In Europe, this species has invaded many types of plant communities (Csiszár et al. 2013; Möllerová 2005). At a young age, it can be classified as moderately shade tolerant, showing the highest growth rate in 25 % full sun, and a slightly lower rate in 100 % (Robakowski, unpublished). In European countries, *P*. *serotina* is considered to be an aggressive colonizer, outcompeting native tree species (Halarewicz 2011).

*Quercus petraea* (Matt.) Liebl. is a deciduous broad-leaved tree. In its natural range, mean annual temperatures are between 5 and 15 °C, mean annual rainfall is 400 to 2500 mm, mean maximum temperature of the hottest month is 10 to 20 °C, and mean minimum temperature of the coldest month is −15 to −8 °C (Forestry Compendium 2005). *Q*uercus *petraea* is classified as shade-tolerant as a juvenile, but its light requirements increase with age (Zarzycki et al. 2002). It tolerates a wide range of soil conditions, but it is best-suited to well-aerated, not too wet, deep, and fertile soils (Modrzyński et al. 2006). In Poland and Germany, *Q*. *petraea* finds favorable conditions for natural regeneration beneath the canopy of pine stands (Kenk 1993). Natural regeneration of *Q*. *petraea* occurs together with the more abundant natural regeneration of *P*. *serotina* under the canopy of *Pinus sylvestris* (L.) stands of different age (Robakowski, personal observation).

### Cultivation of Seedlings

In October 2011, acorns of *Quercus petraea* were collected in a selected seed stand located in the Jarocin Forest Division, Western Poland. In March 2012, before seeding, a small fragment was cut out at the cap side of the acorns to synchronize germination time (Giertych and Suszka 2011). Seeds were sown in containers for germination, and seedlings were grown in peat and perlite (3:1; *v*/v) substrate.

Since *P*. *serotina* is a highly invasive species and therefore not allowed to be cultivated in forest nurseries in Poland, seedlings used in our experiments originated from natural regeneration occurring in large numbers in the “Zielonka” Experimental Forest, 27 km from Poznan (52°33′29″N; 17°06′18″E). At the beginning of May, one-yr.-old seedlings were unearthed, put into plastic boxes, and their roots were covered with humid humus. Seedlings were transported to the Poznan University of Life Sciences Dendrological Garden.

To reduce ontogenetic effects, seedlings of a similar size were selected. *Quercus* and *Prunus* seedlings were potted using 225 7-L pots filled with a mixture of sand, peat, and humus (1/1/1; *v*/v/v). The pH of the substrate was neutral. Before planting, 100 g of fresh *P*. *serotina* leaves, cut into small pieces (~ 0.25 cm^2^), were added to each of 90 pots and mixed with the substrate to enhance the expected allelopathic reaction. Mulching was repeated once a month from May to September with 10 g of freshly cut *P*. *serotina* leaves. In leaves used for mulching, prunasin concentrations were 2.86 ± 048 and 15.9 ± 4.89 mg g^−1^ FW (mean ± SE, FW – fresh weight, *N* = 4) in May and August, respectively. The lowest leaf prunasin concentration was 1.93, and the highest value was 29.93 mg g^−1^ FW. Prunasin was not found in the substrate, even after mulching with *P*. *serotina* leaves. The seedlings were planted using cardboard templates to keep a 5 cm distance between them. In the control group, three *Quercus* seedlings or three *Prunus* seedlings were planted along the central axis of a pot. When grown in interspecific competition, *Quercus* seedlings were planted along the diameter of a pot, and six *Prunus* seedlings were distributed by threes along both sides of the *Quercus* seedlings. Five combinations were obtained using different numbers of seedlings and different mulching scenarios:

1) Q - three seedlings of *Quercus petraea*; 2) P - three seedlings of *P*. *serotina*; 3) Q + L - three seedlings of *Q. petraea* + mulching with *P. serotina* leaves; 4) Q + P - three seedlings of *Q. petraea* + six seedlings of *P*. *serotina*; 5) Q + P + L - three seedlings of *Q. petraea* + six seedlings of *P*. *serotina* + mulching. All combinations are schematically shown in Fig. S1 (Supplementary Material). We focused on two combinations to investigate prunasin concentration in *P*. *serotina* roots: P and Q + P + L.

One week after planting, the seedlings were fertilized using 15 g of the slow-releasing fertilizer ‘Osmocote Exact Standard’ (N, P, K, Mg – 15:9:12:2 and microelements) per pot. Every 2 d, seedlings were watered up to field capacity by using an automatic irrigation system. Watering was less intensive under the low light regime (10 % full light). Seedlings were grown under experimental conditions from mid-May to the end of November.

### Experimental Design

The 225 pots were distributed into three blocks (75 pots per block). Each block was divided into three light treatments: LL (10 %), ML (25 %), and HL (100 % or full irradiance). There were 25 pots in each light treatment. Five combinations of seedlings with or without mulching were distributed in split-plots (five pots per block, light treatment and combination). Each combination was repeated five times in each plot, i.e., 225 experimental units (5 repetitions × 5 combinations × 3 light treatments × 3 blocks =225 pots). We used two combinations, P and Q + P + L (90 pots), to investigate prunasin in *P*. *serotina* roots.

Every month, from June to September, we randomly chose one pot per block, light treatment, and combination to measure structural, morphological, and physiological seedling parameters. The measurements were conducted on two (prunasin concentration in roots, structural parameters, and gas exchange) or three seedlings (biomass allocation and chlorophyll *a* fluorescence) of each species per pot. Every 2 wk., the position of the pots was randomly changed within a block and light treatment to homogenize light conditions.

### Light Treatments

Three different light treatments were established using a shading net; the optical proprieties of the net are described in Wyka et al. (2007). Photosynthetic photon flux (PPF) was measured simultaneously in shading tents above each pot and in the open space by using two light sensors (Spectrum Technologies, Inc., USA). Measurements of PPF were repeated on three occasions on cloudy days in May, July, and September. The relative values of light level were calculated using the following formula: rPPF (%) = (PPF in shade/PPF in the open) × 100.

### Meteorological Conditions

Air temperature and relative humidity (*RH*) were monitored with HOBO Pro v2 (*OnSet Computers*, Pocasset, MA, USA) under the experimental light environments throughout the growing season. Six HOBOs (two per light treatment) were fixed 80 cm above the ground and registered data every twenty min. Monthly mean temperature (*T*_mean_), monthly maximum temperature (*T*_max_), monthly minimum temperature (*T*_min_), and monthly mean *RH* were calculated (Table 1). Microclimatic differences were most noticeable between both shade treatments and HL. Shading decreased monthly mean temperatures and increased monthly amplitudes and *RH* compared to HL (Table 1). The coldest months were May and September, the hottest month was August. The differences between HL and ML in *T*_mean_ were 1.62 in June, 1.71 in August, and 0.97 °C in September. The lowest *T*_min_ and the highest *T*_max_ values were observed in HL.

**Table 1:** Meteorological conditions during the experiment. Mean (±SE) monthly, minimum, maximum monthly temperatures, and mean monthly relative humidity (RH) in three light treatments: 10 % low light (LL), 25 % medium light (ML) or 100 % high light (HL) of full natural sun light

Month	Light treatment (% of full PAR)	Mean monthly temp. (°C)	Minimum monthly temp. (°C)	Maximum monthly temp. (°C)	Mean monthly RH (%)
May	-	15.87 ± 0.15	−0.23	35.08	68.65 ± 0.44
June	10	16.24 ± 0.11	4.06	30.82	80.83 ± 0.39
	25	15.02 ± 0.14	4.04	31.03	82.40 ± 0.39
	100	16.64 ± 0.12	3.49	33.21	80.50 ± 0.39
August	10	17.99 ± 0.11	6.08	35.29	83.80 ± 0.39
	25	17.10 ± 0.11	6.15	35.80	85.64 ± 0.39
	100	18.81 ± 0.13	4.19	35.90	81.66 ± 0.43
September	10	13.63 ± 0.10	2.69	31.56	86.70 ± 0.34
	25	13.35 ± 0.10	2.90	31.38	88.62 ± 0.33
	100	14.32 ± 0.13	1.34	34.81	82.65 ± 0.43

### Morphological Traits

In May, before the beginning of the experiment, the shoot length, (*S*), root length (*R*), the diameter at root collar (*D*), and biomass allocation in ten seedlings of *Q*. *petraea* and ten seedlings of *P*. *serotina* were measured. The results were regarded as an absolute control. Shoot length, root length, and biomass allocation then were measured on five occasions once a month from June to November. The seedlings of the study species were divided into roots, shoots, and leaves, and were weighed. The leaves were scanned with a resolution of 300 dpi, and the leaf projected area was determined using the program “DigiShape” (Cortex Nova, Poland). Plant organs were dried at 65 °C for 48 h (Pol-Eko, Poland), and dry mass (DM) was weighed (Sartorius, Germany).

The parameters describing biomass allocation in a seedling have been defined in Hunt et al. (2002); Portsmuth and Niinemets (2007); Poorter et al. (2012); Reich et al. (1998). Equations for calculating the parameters of biomass allocation are given in Supplementary Material.

### Chlorophyll *a* Fluorescence

Pots with seedlings (one pot per block, light treatment, and combination) were transported to the laboratory and kept in a dark room for 12 h. A fully expanded leaf was selected from the top part and the same exposure of a crown. A leaf-clip was attached to the leaf, and the leaf chamber of the plant efficiency analyzer (PEA, Hansatech, Norfolk, UK) was mounted. The continuous red light of 1 s induced chlorophyll *a* fluorescence. The induction light was 3 200 μmol m^−2^ s^−1^. Basic fluorescence yield (*F*_*0*_) and maximal fluorescence yield (*F*_*m*_) were calculated by the algorithm from fluorescence induction curves, and maximum quantum yield of PSII photochemistry (*F*_*v*_/*F*_*m*_ = *F*_*m*_*- F*_*0*_/*F*_*m*_) (*F*_*v*_ – variable fluorescence) was calculated using the built-in software of the PEA (Operating Instructions for PEA 1999).

### Gas Exchange

Net assimilation of CO_2_ (*A*), leaf transpiration (*E*), and stomatal conductance (*g*_s_) was measured immediately after chlorophyll *a* fluorescence on the same leaf by using the gas exchange analyzer LCA-4 (ADC, Ltd., Hoddesdon, UK) equipped with the broadleaf chamber (PLC4B). Air flow was 138 μmol s^−1^, and CO_2_ concentration in inlet air was 380 μmol mol^−1^. Leaf temperature was controlled by thermocouple and ranged from 26 to 27 °C. Relative air humidity in a leaf chamber was approximately 55 %. To stabilize air humidity in the leaf chamber, inlet air was enriched with water vapor by using an air humidifier (CF-2658, Comefresh Electronic Industry, Co. Ltd., China). Light was provided by a diode lamp (Parathom, PAR, day light, 4.5 W, Osram). Gas exchange was measured at the saturation light level of 1200 μmol m^−2^ s^−1^. The leaf was placed in the leaf chamber and was given 20 to 30 min to acclimate to the leaf chamber conditions. The window of the leaf chamber was darkened, and dark respiration (*R*_d_) was measured for 15 min. The lamp then was switched on, and gas exchange was measured for 30 min. Data were registered every min to generate a curve of photosynthesis from the induction to a stable saturation level. Five values from the stable phase of a curve were used to calculate maximum net assimilation rate (*A*_max_). Water use efficiency was calculated as a ratio of *A*_max_ to *E*. *A*_max_ was expressed in μmol m^−2^ s^−1^ and nmol g^−1^ DM s^−1^. Nitrogen concentration was determined in the same leaves as net CO_2_ assimilation rate to calculate photosynthetic nitrogen use efficiency (*PNUE*, μmol CO_2_ mol *N*^−1^ s^−1^).

### Prunasin Concentration in Roots of *Prunus serotina*

Prunasin concentration in roots of the *P*. *serotina* seedlings was determined using high performance liquid chromatography (HPLC) (De Nicola et al. 2011; Vetter 2000). To avoid possible interference of the results caused by tannins and plant pigments in the matrix, active carbon was added into the extract as scavenger (Berenguer-Navarro et al. 2002).

### Sampling of Plant Material

Seedlings were removed from pots and their roots were thoroughly washed with tap water. In the laboratory, the main root was measured and at the midway point, a fragment of the root was cut and weighed. Fresh mass of root samples was 200 mg. Samples were put into pre-cooled Eppendorf tubes and stored at −25 °C. Root samples for prunasin concentration analyses were collected on four occasions: in May before planting and during the experiment at the end of June, July, and September. The total number of samples was ten absolute control + three repetitions in time x three blocks x three light treatments x two combinations of competition x two seedlings per pot =118.

### Prunasin Extraction

Root samples of 200 mg were shaken with 10 ml methanol for approximately 10 h at room temperature. Carbon was activated at 200 °C and kept in a desiccator. Then, 0.5 g of activated carbon were added to leaf extracts, and mixtures were centrifuged at 3000 rpm for 10 min. The supernatant was filtered through a 0.45 μm nylon filter. Aliquots were injected directly into the column.

### HPLC

Analyses were conducted with Separations Module 2695 (Waters, USA), using the U*V*/Visible 2489 detector (Waters). Determination of prunasin was performed isocratically using the SunFireTM C18 column (4.6 × 250 mm, 5 μm) together with the pre-column Waters (4.6 × 20 mm). The temperature of the column was stabilized at 40 °C. The methanol: water (15: 85 *v*/v) solution was used as eluent at 1.5 ml/min flow rate, and UV detection was set at 218 nm.

The concentration of prunasin in extracts was calculated from the calibration curve obtained using commercially available chromatographically purified prunasin (ABCR GmbH & Co. KG, Karlsruhe, Germany) as a standard. Prunasin concentration was first expressed as mg g^−1^ root fresh mass, and it was recalculated using fresh weight to dry weight root ratio and expressed as mg g^−1^ DM.

### Nutrient Concentration

Nitrogen, phosphorous, potassium, calcium, and magnesium concentrations were determined using the same leaves on which chlorophyll *a* fluorescence and gas exchange had been measured. Leaves were sampled on four occasions: in May (absolute control at the start of the experiment), June, August, and September. Total nitrogen content was determined using the Kjeldahl method. The digestion of leaf samples was conducted with a digestion system with sulfuric acid at 420 °C (Foss Tecator). Nitrogen was determined by distillation with water vapor in a Parnas-Wagner apparatus. For determination of P, K, Mg, and Ca, leaf samples were combusted at 500 °C for 8 h. The ash was dissolved in HCl, and the solution was used for further analyses. Phosphorous concentration in leaves was determined using the spectrophotometric molybdovanadate method. Absorbance was measured using a Specol 21 spectrophotometer (Carl Zeiss Jena, Germany) and compared with the calibration curve obtained from the standards. Magnesium was determined with Atomic Absorption Spectrometry using AAS 1 N (Carl Zeiss Jena, Germany). Photoelectric flame photometry (FlaPho 40, Carl Zeiss Jena, Germany) was applied to determine potassium and calcium concentrations. All nutrient analyses in leaves were conducted at the Department of Chemistry, Poznan University of Life Sciences, Poland.

### Statistical Analyses

Before analysis, the data were tested for normality and homogeneity of variance. When necessary, data were logarithmically transformed [(*z* = log_10_(*y* + 1)] or the function $z=\mathit{\arcsin}\sqrt{p}$was applied to obtain normal distribution. The effects of sampling time, light, and combination on prunasin in *P*. *serotina* roots was tested using analysis of variance in split-split plot design. This experimental design is also called “split-split plot in time” (Qiunn and Keough 2002). The linear model of ANOVA in split-split plot design is described in Supplementary Material.

To determine the relationship between morphological, architectural, structural, and physiological parameters and prunasin concentration in roots of *P. serotina*, we used analysis of correlation. Pearson’s coefficients of correlation (*r*) with probabilities were calculated. Additionally, linear regression between the measured traits and prunasin concentration was analyzed in *P. serotina* seedlings growing in one of two combinations: P or Q + P + L under high light. All statistical analyses were conducted using Statistica 10.0 (StatSoft, Inc., Tulsa, USA) and Sigmaplot 13.0.

## Results

### Effects of Microclimatic Conditions and Competition on Prunasin Concentration

**Fig. 1:**
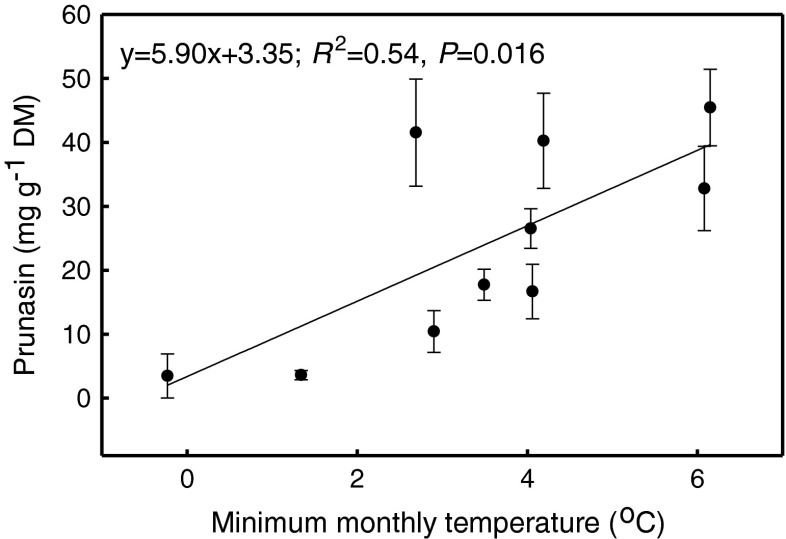
The relationship between the minimum monthly temperature and mean prunasin concentrations (means ± SE) in roots of *Prunus serotina*. Prunasin concentration was determined in May, June, August, and September. The equation of linear regression, coefficient of determination (*R* ^2^) with probability (*P*) obtained from the analysis of variance in regression are given (*N* = 10, *N* – pairs of the values of minimum monthly temperature and mean prunasin concentration per light treatment)

Prunasin concentration in *P*. *serotina* roots was determined on four occasions under different conditions of air temperature and humidity (Table 1). The lowest mean prunasin concentration was in May. Prunasin in June was lower than in August, but did not differ significantly from September in split-split plot ANOVA and Tukey’s test (Tables 2 and 3). When compared with May, prunasin increased eleven fold in August and approximately six fold in June and September (Table 3). Concentration was positively correlated with *T*_min_, with *T*_min_, explaining 54 % of the variation (Fig. 1).

**Table 2:** Analysis of variance for prunasin concentration in roots of *Prunus serotina* seedlings acclimated to one of three light regimes: 10 % low light (LL), 25 % medium light (ML), or 100 % high light (HL) of full natural sun light. Samples were taken on three occasions: in June (DOY 176, DOY – day of year), in August (DOY 238), and in September (DOY 273). The split-split plot model of ANOVA was applied with block, time of sampling, light treatment, combination, and interactions as the sources of variance. The differences were significant at *P* ≤ 0.05. MS – mean sum of squares, *F* – value of Snedecor’s function, in bold – statistically significant at *P* < 0.05 (*N* = 106, *N* – number of seedlings)

Source of variance	Effect	Degrees of freedom	MS	*F*	*P*
Block	Random	2	0.22	0.21	0.065
Time of sampling	Fixed	2	**28.70**	**26.95**	**0.005**
Error I (Block × Time of sampling)	Random	4	1.07	0.67	0.626
Light	Fixed	2	**6.42**	**4.03**	**0.045**
Time of sampling × Light	Fixed	4	**13.58**	**8.53**	**0.002**
Error II (Block × Light + Block × Time × Light)	Random	12	1.59	1.13	0.401
Competition with mulching	Fixed	1	0.27	0.19	0.666
Time of sampling × Competition with mulching	Fixed	2	0.58	0.411	0.669
Light × Competition with mulching	Fixed	2	2.01	1.42	0.269
Time of sampling × Light × Competition with mul.	Fixed	4	3.55	2.51	0.081
Error III	Random	17	1.41		
Total		52

**Table 3:** Mean (±SE) concentration of prunasin in roots expressed per root dry mass of *Prunus serotina* seedlings in function of time, light treatments, combinations of seedlings, and mulching, and interactions. The different letters indicate that the mean values are significantly different in Tukey’s a posteriori test at α = 0.05 (α – level of significance). DOY – day of year, LL - low light (10 %), ML - medium light (25 %), HL - high light (100 %), P – three *P*. *serotina* seedlings (monoculture), Q + P + L – three *Quercus petraea* + six *P*. *serotina* + mulching with *P*. *serotina* leaves

Effects		*N*	Mean ± SE (mg g^−1^ DM)	
Control	May	9	3.45 ± 0.52	
Time of sampling	June (DOY 176)	34	19.95 ± 2.12	a
	August (DOY 238)	36	39.50 ± 3.85	b
	September (DOY 269)	36	18.51 ± 4.90	a
Light	LL	36	30.33 ± 4.37	a
	ML	34	27.52 ± 4.41	ab
	HL	36	20.50 ± 4.41	b
Competition	P	52	26.02 ± 3.88	
	Q + P + L	54	26.18 ± 3.42	
Time of sampling × light	176 × LL	12	16.68 ± 4.24	a
	176 × ML	10	26.51 ± 3.10	a
	176 × HL	12	17.74 ± 2.42	a
	238 × LL	12	32.79 ± 6.60	a
	238 × ML	12	45.45 ± 5.98	a
	238 × HL	12	40.25 ± 7.43	a
	269 × LL	12	41.53 ± 8.36	a
	269 × ML	12	10.42 ± 3.28	b
	269 × HL	12	3.57 ± 0.73	b
Time of sampling × competition	177 × P	16	21.28 ± 3.38	
	177 × Q + P + L	18	18.77 ± 2.77	
	238 × P	18	36.60 ± 6.32	
	238 × Q + P + L	18	42.40 ± 4.57	
	269 × P	18	19.65 ± 8.10	
	269 × Q + P + L	18	17.37 ± 6.01	
Light × competition	LL × P	18	30.02 ± 6.57	
	LL × Q + P + L	18	30.65 ± 6.17	
	ML × P	16	24.66 ± 7.64	
	ML × Q + P + L	18	30.06 ± 5.12	
	HL × P	18	23.23 ± 6.65	
	HL × Q + P + L	18	17.82 ± 6.06

Prunasin concentration differed among the seedlings acclimated to different light environments. The highest concentration was found in plants grown in LL, which differed from those in HL, but not in ML (Tables 2 and 3). The significant interaction between sampling time and light indicates that the effect of sampling time was modified by the light conditions. Prunasin was highest in LL compared with ML and HL in September (Table 3). When the data from all light treatments were pooled, there were no differences in prunasin concentration between *P*. *serotina* seedlings growing in a monoculture and Q + P + L. The interactions between the sampling time or light and combination were not significant (Table 2). Mean root DM in HL was approximately nine times higher than in LL and 2.7 times higher in ML. Thus, when prunasin concentrations expressed per mean root DM were compared among light treatments, the highest amount of was produced in HL (31.3 ± 18.7, 15.4 ± 10.8, and 7.8 ± 3.9 mg g^−1^ root DM in HL, ML, and LL, respectively), suggesting that the differences among the light treatments are due, at least partially, to a shift in ontogenetic development.

### Correlation between Biometrical Parameters and Prunasin

Pooling of all data led to an increase of prunasin with declining root weight ratio (*RWR*) (*R*^2^ = 0.32, *P* < 0.001). In LL, the monoculture, and Q + P + L, there were no correlations between morphological or architectural parameters of *P*. *serotina* seedlings and prunasin concentration (Table 4). A decrease in prunasin was correlated with increasing *RWR* in both ML and HL, and with leaf mass to area ratio (*LMA*) in HL. In contrast, prunasin decreased with shoot length to shoot diameter at root collar ratio (*SL*/*SD*) in ML and root dry weight (*W*_*R*_) in HL. Prunasin concentration was positively related to root to shoot ratio (*R*/*S*), leaf weight ratio (*LWR*), and leaf area ratio (*LAR*) in both shade treatments, and to *LMA* in ML (Table 4).

**Table 4:** Correlation between prunasin concentration in roots and morphological and architectural parameters of *Prunus serotina* seedlings growing in 10 % low light (LL), 25 % medium light (ML), or 100 % high light (HL) of full natural sun light and in one of two combinations of seedlings: P (three *P*. *serotina* seedlings) or Q + P + L (three *Quercus petraea* + six *P*. *serotina* + mulching with *P*. *serotina* leaves). All data from three sampling dates (June, August, and September) were analyzed. *RL* – root length, *SL* – shoot length, *SD* – shoot diameter at root collar, *RLR* – root length ratio, *W*_*R*_ – root dry weight, *W*_*S*_ – shoot dry weight, *W*_*L*_ – leaves dry weight, *W* – total seedling dry weight, *A*_*L*_ – leaf area, *R*/*S* – root: shoot ratio, *RWR* – root weight ratio, *SWR* – shoot weight ratio, *LWR* – leaf weight ratio, *LAR* – leaf area ratio, *LMA* – leaf mass to area ratio. The definitions of the calculated parameters are given in Supplementary Material. *r* – Pearson’s coefficient of correlation, ^*^0.01 ≤ *P* < 0.05, ^**^0.001 ≤ *P* < 0.01, ^***^ *P* < 0.001, in bold – *r* statistically significant (*N* = 17)

Morphological and architectural parameters	Pearson’s coefficient of correlation prunasin vs. parameter		
	LL	ML	HL
RL (mm)	0.373	−0.245	−0.227
SL (mm)	0.080	0.217	−0.104
SD (mm)	−0.158	−0.134	−0.316
SL/SD	−0.119	**−0.700** ^**^	−0.378
RLR (m g^−1^)	−0.132	0.142	0.245
W_R_ (g)	0.154	−0.460	**−0.615** ^**^
W_S_ (g)	0.275	−0.131	−0.431
W_L_ (g)	0.187	−0.166	−0.370
W (g)	0.208	−0.258	−0.445
A_L_ (m^2^)	0.221	−0.055	−0.199
R/S	0.096	**0.657** ^**^	**0.804** ^***^
RWR	−0.173	**−0.735** ^**^	**−0.827** ^***^
SWR	−0.139	0.304	0.324
LWR	0.261	**0.533** ^*^	**0.723** ^**^
LAR (m^2^ g)	0.055	**0.690** ^**^	**0.777** ^***^
LMA (g m^−2^)	0.467	**0.538** ^*^	**−0.564** ^*^

### Relationships between Physiological Characteristics and Prunasin

Significant correlations between physiological parameters and prunasin concentration were found mainly in the shade treatments. In HL, only a decrease in prunasin with greater *WUE* (water use efficiency) was significant (Table 5). In LL, prunasin concentration was positively correlated with nitrogen content per unit leaf area, but not when N was expressed per unit leaf dry mass. Prunasin was negatively correlated with *F*_*v*_/*F*_*m*_ and *E*. In ML, the important photosynthetic parameters (*A*_max_ and *PNUE*) increased with higher prunasin concentrations (Table 5).

**Table 5:** Correlation between prunasin concentration in roots and structural, and physiological parameters of *Prunus serotina* seedlings growing in 10 % low light (LL), 25 % medium light (ML), or 100 % high light (HL) of full natural sun light and in one of two competition treatment: *P* (three *P*. *serotina* seedlings) or Q + P + L (three *Quercus petraea* + six *P*. *serotina* + mulching with *P*. leaves). All data from three sampling dates (June, August, and September) were pooled. *Chl tot* – total chlorophyll content in leaf, *N* – nitrogen content in leaf, *F*_*v*_/*F*_*m*_ – maximum quantum yield of PSII photochemistry, *R*_*d.*_ – dark respiration, *A*_max_ – maximum net CO_2_ assimilation rate, *E* – transpiration rate, *WUE* – water use efficiency, *PNUE* – photosynthetic nitrogen use efficiency. *r* – Pearson’s coefficient of correlation, ^*^0.01 ≤ *P* < 0.05, ^**^0.001 ≤ *P* < 0.01, ^***^ *P* < 0.001, in bold – *r* statistically significant (*N* = 17)

Structural and physiological parameters	Pearson’s coefficient of correlation		
	LL	ML	HL
Chl tot (mg m^−2^)	−0.389	−0.253	−0.429
N (mg g^−1^)	0.314	0.187	0.196
N (g m^−2^)	**0.530** ^*^	−0.229	0.215
F_v_/F_m_	**−0.609** ^*^	0.170	0.355
R_d_ (μmol m^−2^ s^−1^)	−0.098	−0.169	−0.322
R_d_ (nmol g^−1^ s^−1^)	0.131	−0.426	−0.370
A_max_ (μmol m^−2^ s^−1^)	−0.416	**0.706** ^**^	0.338
A_max_ (nmol g^−1^ s^−1^)	−0.299	**0.575** ^*^	0.104
E (mmol m^−2^ s^−1^)	**−0.510** ^*^	**0.541** ^*^	0.491
WUE (μmol mmol^−1^)	**0.510** ^*^	−0.241	**−0.680** ^**^
PNUE (μmol mmol^−1^)	−0.378	**0.591** ^*^	0.380

### Effects of Competition with Mulching on Prunasin Concentration

**Fig. 2:**
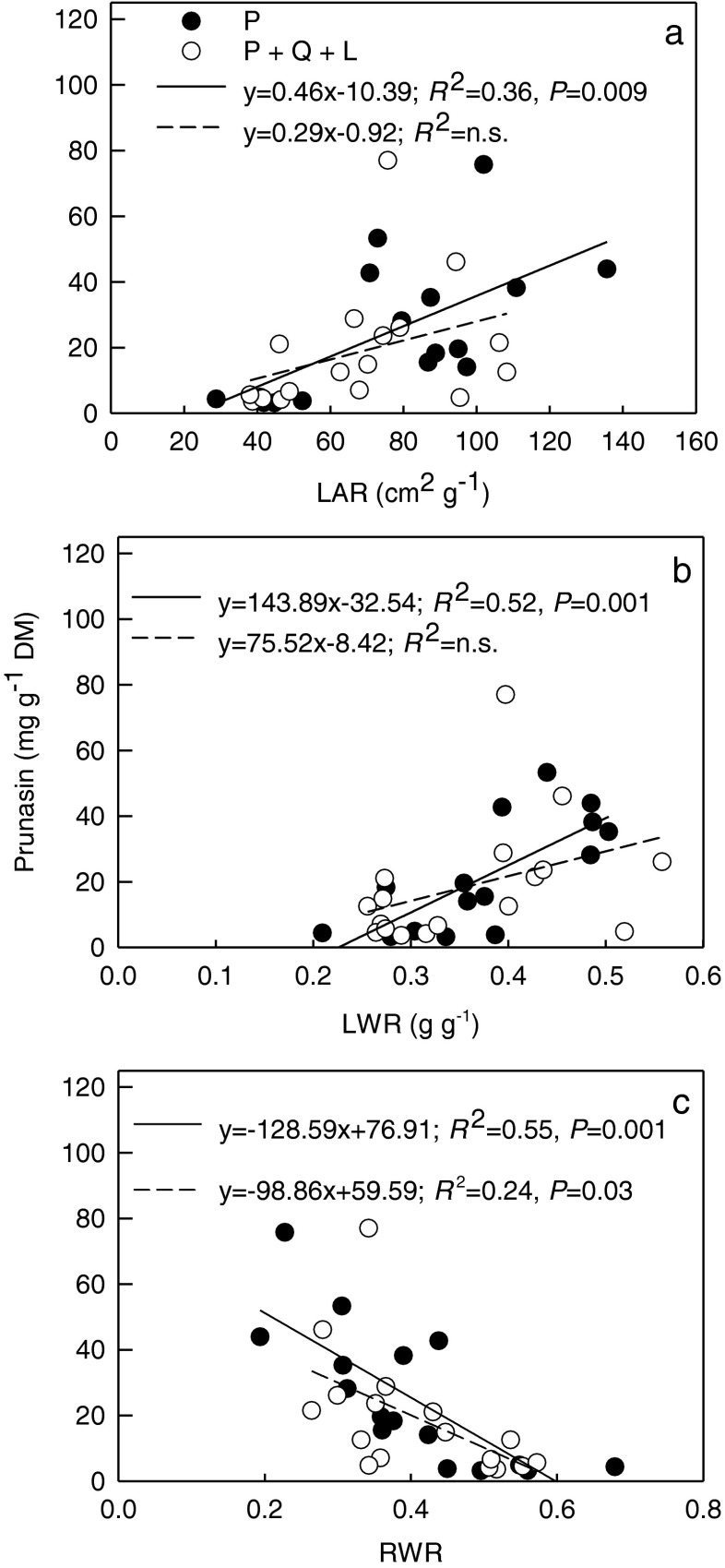
The linear regression between the architectural parameters and prunasin concentration in roots of *Prunus serotina* seedlings growing in control (P – three *Prunus* seedlings) or in competition with mulching (Q + P + L - three *Quercus petraea* + six *P*. *serotina* + mulching with *P*. *serotina* leaves) under high light (HL): **a** Leaf area ratio (*LAR*); **b** Leaf weight ratio (*LWR*); **c** Root weight ratio (*RWR*) vs. prunasin concentration in *P*. *serotina* roots. The equations of linear regression, coefficients of determination (*R* ^2^) with probability (*P*) obtained from the analysis of variance in regression are shown (*N* = 16; *N* – number of seedlings)

In HL, the relationships between *LAR*, *LWR*, and root prunasin were significant for *P*. *serotina* seedlings growing in monoculture, but this was not the case in Q + P + L (competition with mulching) (Fig. 2a, b). Only with *RWR*, a decrease in prunasin concentration was significant in both treatments (Fig. 2c).

**Fig. 3:**
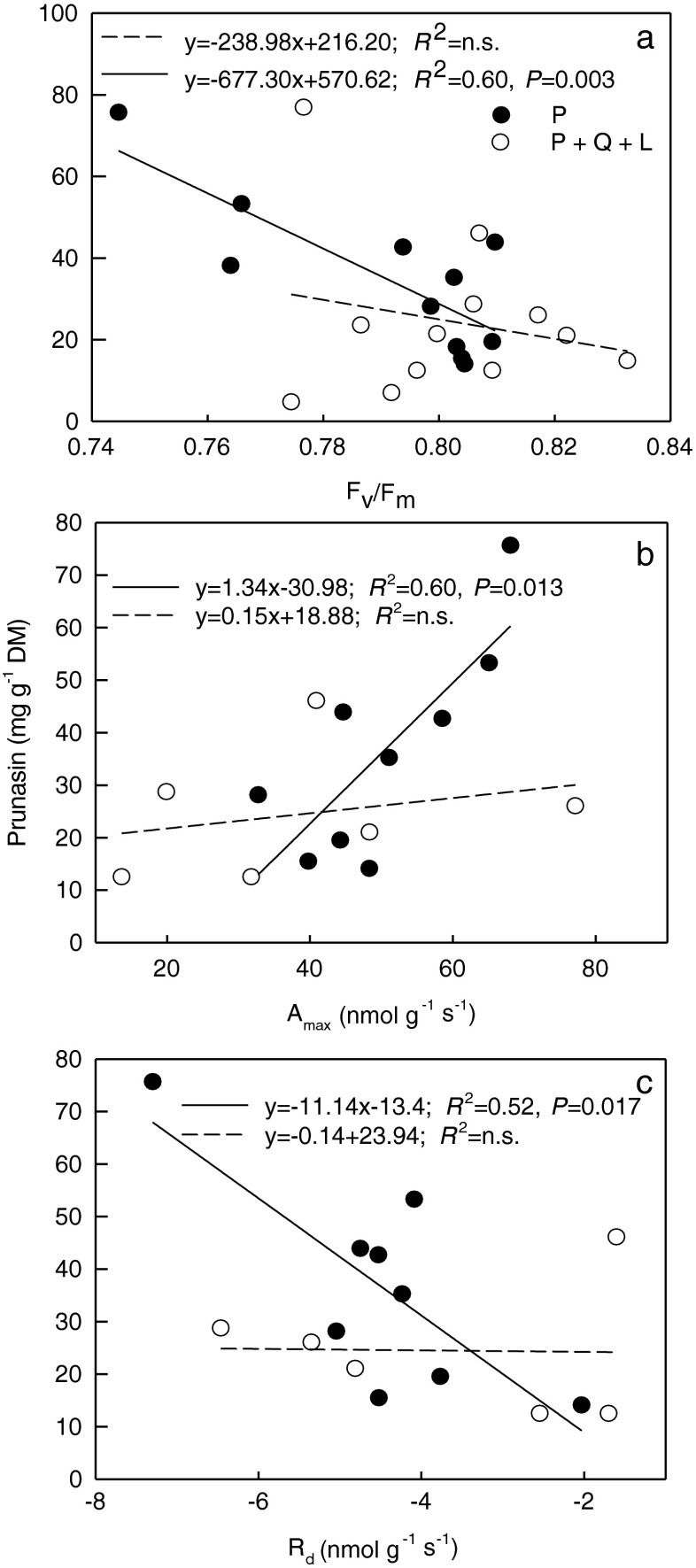
The linear regression between the photosynthetic parameters and prunasin concentration in roots of *Prunus serotina* seedlings growing in control (P – three *Prunus* seedlings) or in competition with mulching (Q + P + L - three *Quercus petraea* + six *P*. *serotina* + mulching with *P*. *serotina* leaves) under high light: a. Maximum quantum yield of PS II photochemistry (*F*_*v*_/*F*_*m*_); b. Maximum net CO_2_ assimilation rate (*A*_max_); c. Dark respiration (*R*_d_) vs. prunasin concentration in *Prunus serotina* roots. The equations of linear regression, coefficients of determination (*R* ^2^) with probability (*P*) obtained from the analysis of variance in regression are shown (*N* = 9 − 11)

Prunasin decreased with increasing *F*_*v*_/*F*_*m*_ in monoculture, but this trend was not significant in Q + P + L (Fig. 3a). The relationship between *A*_max_ expressed per unit leaf dry mass and prunasin concentration was positive in monoculture, but not significant in Q + P + L (Fig. 3b). Root prunasin decreased with declining *R*_d._ in monoculture and was nearly stable in Q + P + L (Fig. 3c).

**Fig. 4:**
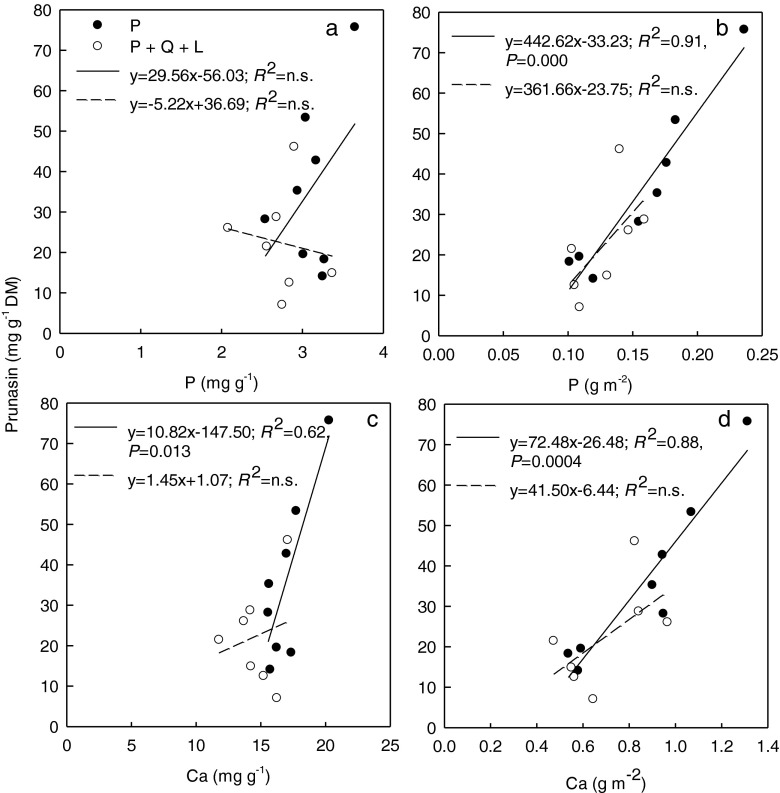
The linear regression between phosphorous (P) or calcium (Ca) content in leaves and prunasin concentration in roots of *Prunus serotina* seedlings growing in control (P – three *Prunus* seedlings) or in competition with mulching (Q + P + L - three *Quercus petraea* + six *P*. *serotina* + mulching with *P*. leaves) under high light: **a** Phosphorous concentration in leaves expressed per leaf dry mass vs. prunasin concentration; **b** Phosphorous content per leaf area vs. prunasin concentration; **c** Calcium concentration per leaf dry mass vs. prunasin concentration; **d** Calcium content per leaf area vs. prunasin concentration in *Prunus* roots. The equations of linear regression, coefficients of determination (*R* ^2^) with probability (*P*) obtained from the analysis of variance in regression are shown (*N* = 8)

Relationships between phosphorous, calcium, and root prunasin depended on the expression of their content per unit leaf dry mass or leaf area (Fig. 4a, b, c, d). Phosphorous content per DM increased linearly when expressed per unit leaf area in monoculture (Fig. 4b). In monoculture, leaf calcium content was positively related to root prunasin concentration, independently of being expressed per leaf DM or area, but not in Q + P + L (Fig. 4c, d).

## Discussion

### Seasonal Variation in Prunasin Concentration

In roots of one-yr.-old *P*. *serotina* seedlings, prunasin varied during the vegetative season and was influenced by light conditions. In May, at the early stage of seedling development, concentration was lowest. It increased up to a maximum value in August, concurrent with air temperature and *P. serotina* growth rate. It declined in September, except for the seedlings growing in LL (Table 3). Results obtained in June in ML and HL and in September in ML were similar to those of Graham (2002), who reported prunasin concentrations of 12.8 to 14.5 mg g^−1^ DM in roots of Nemaguard peach seedlings grown in a greenhouse. In August, however, prunasin in roots was two to three times higher than in peaches. Around the same time, Miller et al. (2004) documented a mean prunasin concentration of 21.78 mg g^−1^ DM in roots of *Prunus turneriana* (F.M.Bailey) Kalman, which is closely phylogenetically related to *P. serotina.*

In the present study, prunasin decreased exponentially with increasing water content in roots (*R*^2^ = 0.3, *P* < 0.001) and was negatively correlated with photosynthetic water use efficiency in HL (Table 5). This is in agreement with earlier studies showing a decrease in root concentrations in *Prunus dulcis* Mill. and an increase in HCN release in lima bean grown under restricted water supply (Arzani et al. 2010; Ballhorn et al. 2011b).

Our study is the first to determine prunasin concentration in *P*. *serotina* roots on several occasions during the vegetative season. The course of changes was, to some extent, consistent with the results of earlier studies. This indicates that the amount of prunasin in leaves, twigs, and fruits depends on sampling time, plant developmental stage, and environmental conditions (Gleadow and Woodrow 2000; Neilson et al. 2013; Swain et al. 1992). Similar to our results, prunasin concentration in twigs and leaves of *Amelanchier alnifolia* (Nutt.) also varied seasonally (Majak et al. 1980). However, in *A*. *alnifolia*, prunasin decreased at the start of May and stabilized in July and August with diminishing intensity of photosynthesis.

Prunasin concentration also varied seasonally in young leaf tips of field-grown *Eucalyptus cladocalyx* (F. Muell.); seasonal variation was correlated with changes in leaf nitrogen content per unit leaf area (Gleadow and Woodrow 2000). In our study, prunasin root concentration was only weakly correlated with leaf nitrogen content expressed per unit leaf area in LL, where some trade-off was expected between investment of resources in defense against herbivores and light harvesting protein-pigment complexes (Neilson et al. 2013; Niinemets 1998). Root prunasin also has been positively correlated with leaf nitrogen in *Prunus dulcis* (Arzani et al. 2010).

In our study, prunasin concentration was linearly positively correlated with minimal monthly temperatures measured above the pots. Similarly, in *Trifolium repens* (L.), a positive correlation was found between total cyanogen content and mean air temperature for four days before sampling (Stochmal and Oleszek 1997). The results of Stochmal and Oleszek (1997) and our results suggest also that synthesis of cyanogenic glycosides increases in a warming climate. Thus, we hypothesize that these compounds, including prunasin, may not only play the role of an effective deterrent against insects or browsers when present in roots, twigs, and leaves (Ballhorn 2011; Patton et al. 1997), but also of a carbon store (Selmar et al. 1988). However, in leaves of cyanogenic *Eucalyptus cladyocalyx*, prunasin did not increase in elevated CO_2_ treatments (Gleadow et al. 1998).

In contrast, due to cyanogenesis, cyanogenic glycosides may become a source of carbon and nitrogen that can be reused in primary metabolism. In particular, such resources are translocated to photosynthetic processes and invested into photosynthetic structures contributing to an increase in CO_2_ uptake (Neilson et al. 2013). Cyanogenic glycosides synthesized in plant organs participate in carbon sequestration (Selmar et al. 1988). Plants may allocate a considerable amount of leaf nitrogen to prunasin - e.g., up to 20 % in leaves of *Eucalyptus cladocalyx* (Gleadow and Woodrow 2000). Changes in concentrations of cyanogenic glycosides affect the balance of carbon and nitrogen in a plant, and may take part in carbon and nitrogen cycles as their sink or source.

### Light Effect on Prunasin Concentration

When prunasin concentration in *P*. *serotina* roots was compared among experimental light environments, the highest values were found in LL, suggesting that it may protect roots of seedlings against insects and nematodes more effectively under shaded conditions. This result is consistent with the findings that shade-acclimated plants are more severely threatened by insects and might invest more resources into specialized metabolites with deterring properties, such as phenols and tannins (Karolewski et al. 2010, 2013). Here, however, the light effect was strongly influenced by sampling time (Table 2). This interaction was even more significant (*P* = 0.002) than the light effect itself (*P* = 0.045), indicating that when growth is inhibited in LL, prunasin synthesis is not reduced. Thus, higher prunasin concentrations in LL than in HL may be due to the fact that it is concentrated in a smaller root mass compared to HL. In our study, a shift in ontogenetic development affected, at least to some extent, the observed differences in root prunasin concentrations among light treatments.

Correlations between prunasin concentration in *P*. *serotina* roots and some morphological and architectural parameters depended on the light environment and were not observed in LL, when growth of seedlings was substantially inhibited (Table 4). For the most part, structural and physiological parameters correlated with prunasin concentration in LL and ML (Table 5). According to resource allocation theory, when shading inhibits growth more than it decreases photosynthesis, *P*. *serotina* seedlings should invest more resources into prunasin and structural and physiological processes (Table 5) (Matyssek et al. 2005). In contrast, however, Burns et al. (2002) showed that in LL, prunasin concentration in *Eucalyptus cladocalyx* leaf was lower than in HL. Additionally, those authors concluded that the slope of linear regression between total leaf nitrogen concentration and leaf cyanide concentration depends on light conditions, suggesting that differences in prunasin concentration can be explained by the optimal resources allocation hypothesis.

In our study, the lower prunasin concentration in *P. serotina* roots in HL likely results from the allocation of resources to photosynthetic processes and growth. Additionally, in HL, nitrogen and carbon may be translocated into the xanthophyll cycle or other mechanisms that protect against photoinhibition. These observations are supported to some extent by Jørgensen et al. (2005), who demonstrated that in cassava, cyanogenic glucosides are transported from the shoots to the roots. Additionally, Gleadow and Woodrow (2000) have shown a significant decrease in the proportion of nitrogen allocated to cyanogenic glucosides in young *Eucalyptus cladocalyx* leaves, coinciding with the peak flowering period. This implies that nitrogen from cyanogenic glycosides is transferred from young leaves to reproductive organs.

According to the optimal resources allocation hypothesis (McKey 1974), high prunasin concentration in *P*. *serotina* roots in LL is determined by two factors: (1) the cost for *P. serotina* seedlings of herbivore-inflicting damage or root loss, and (2) the probability that the roots would be successfully attacked in the absence or low concentration of prunasin. Our results suggest that under LL, photosynthetic gain can be maximized by the reallocation of nitrogen from root defense to the photosynthetic system. However, in LL, an increase in root prunasin occurred at the cost of maximal quantum yield of PSII photochemistry. In contrast to our study, Miller et al. (2004) found no difference in cyanogenic glycoside concentration nor the proportion of nitrogen allocated to cyanogenic glycoside in foliage, stems, or roots of *Prunus turneriana* (F.M.Bailey) Kalkman when comparing plants grown in one of three light treatments.

### Effects of Interspecific Competition on Prunasin Concentration

According to some studies, plants rich in prunasin are more competitive than others species (Badri and Vivanco 2009; Bais et al. 2004, 2006). Hypothetically, in the presence of a competitive species, *Q*. *petraea*, prunasin in roots of invasive *P*. *serotina* acts as a “novel weapon” (Kim and Lee 2010). Here, prunasin levels were similar in the monoculture and competition treatments (Table 2). This result indicates that synthesis of prunasin in *P*. *serotina* roots was not stimulated by interspecific competition. Nevertheless, in *Prunus* monocultures growing in HL, prunasin concentrations were linearly correlated with growth, biomass allocation, and some physiological parameters of *P*. *serotina* seedlings. However, such relationships were not found in *Prunus* seedlings growing in Q + P + L (Figs. 2 and 3). In the HL treatment only, the presence of *Q*. *petraea* influenced the relationships between the measured features and prunasin concentration when compared with *P*. *serotina* seedlings grown in monoculture. In other words, competition with *Q*. *petraea* did not directly affect the quantity of prunasin in roots of *P*. *serotina* seedlings, but influenced the relationships between prunasin concentration and morphological and physiological features modulated by light conditions. This suggests that *P. serotina* growing in interspecific competition utilize a different strategy for prunasin use compared to *P. serotina* growing in monocultures. This difference can be explained by the hypothesis that the costs of reallocating prunasin from roots to leaves, structural traits, and photosynthetic capacity are probably different in *P*. *serotina* growing in monocultures than seedlings growing in the interspecific competition treatment where prunasin concentration was not correlated with these parameters,thus suggesting that an investment of resources into competition with *Q*. *petraea* occurred at the expense of reallocating nitrogen from prunasin to growth and photosynthesis. Still, there is a need to re-evaluate resource allocation-based hypotheses regarding prunasin costs and to consider how multifunctional attributes of prunasin may lower the cost of interspecific competition (Neilson et al. 2013).

Our study emphasizes that variation in prunasin concentration is the result of species-specific adaptation, microclimatic conditions, a shift in ontogenetic development, and, to a lesser extent, interspecific plant-plant interactions. The effect of sampling time, which integrated the effects of changing microclimatic conditions and ontogenetically determined growth dynamics of *P*. *serotina* seedlings, seemed to be the most pronounced one.

Under LL, nitrogen in excess of growth and photosynthetic requirements seems to be allocated to prunasin in *P*. *serotina* roots, and this increase in prunasin would in turn lead to a decrease in *F*_*v*_/*F*_*m*_ (Table 5). Inversely, under ML and HL, relatively more nitrogen was invested in biomass production, photosynthesis, and photoprotection (in HL) at the expense of prunasin synthesis. Interestingly, in ML, an increase in prunasin concentration was associated with higher net CO_2_ assimilation rates and *PNUE*, which suggests a trade-off between increasing *A*_max_ due to higher *PNUE* and greater prunasin concentration (Table 4). In *Eucalytptus cladocalyx*, there was an approximately proportional increase in cyanogenic glycoside concentration with leaf nitrogen concentration (Gleadow et al. 1998). However, Goodger et al. (2004) did not find a significant increase in prunasin when *E*. *polyanthemos* seedlings were grown at high soil nitrogen availability. In our study, where the seedlings were fertilized, relations between prunasin concentration, phosphorous, and calcium were driven by light regimes and, to a lesser extent, by competitive interactions (Fig. 4).

Selmar et al. (1988) demonstrated that linamarin, and possibly other cyanogenic glycosides, serve in the metabolism of developing plants as N-storage compounds. The behavior of *P*. *serotina* in LL indicates that it is an opportunistic species that can store nitrogen in the form of prunasin even in deep shade where its growth is limited. Under more favorable light conditions, the resources stored in root prunasin might be transformed and transported to leaves and reused for photosynthesis and biomass production. This is in agreement with similar studies showing that *P*. *serotina* seedlings initially grow slowly under a dense canopy of young Scots pine forest, while their growth is seven times faster under a more open canopy (Robakowski and Bielinis 2012). In *P. serotina*, prunasin may effect ecological plasticity and invasiveness in response to climate change and competition. Unraveling the coexisting physiological, genetic, and ecological mechanisms influencing prunasin concentration in *P. serotina* roots remains a significant future challenge.

## Electronic supplementary material

ESM 1. The schematic presentation of all combinations of seedlings growing in monoculture or in interspecific competition with or without mulching is shown in Figure S1. In Material and methods there are the equations used to calculate the parameters describing biomass allocation to different organs of a seedling. The model of analysis of variance in split-split plot design is presented in details. (DOCX 20 kb)
