# Supplementary material for: Seasonal Changes Affect Root Prunasin Concentration in Prunus serotina and Override Species Interactions between P. serotina and Quercus petraea
Source: J Chem Ecol. 2016 Mar 9;42:202–14. doi: 10.1007/s10886-016-0678-y (PMC4839042; doi:10.1007/s10886-016-0678-y)
Supplement: Supplementary file 1 — The schematic presentation of all combinations of seedlings growing in monoculture or in interspecific competition with or without mulching is shown in Figure S1. In Material and methods there are the equations used to calculate the parameters describing biomass allocation to different organs of a seedling. The model of analysis of variance in split-split plot design is presented in details. (DOCX 20 kb) [file 10886_2016_678_MOESM1_ESM.docx]

Supplementary Material

Seasonal changes affect root prunasin concentration in *Prunus Serotina* and override species interactions between *P. serotina* and *Quercus petraea*

^1*^Piotr Robakowski, ^1^Ernest Bielinis, ^2^Jerzy Stachowiak, ^3^Iwona Mejza, ^1^Bartosz Bułaj

*^1^Poznan University of Life Sciences, Department of Forestry, Wojska Polskiego 71E St., 60-625 Poznan, Poland; phone: +48 61 848 77 38, fax: +48 61 848 77 34*

*^2^ Poznan University of Life Sciences, Department of Chemistry, Wojska Polskiego 75 St., 60-625 Poznan, Poland*

*^3^Poznan University of Life Sciences, Department of Mathematical and Statistical Methods, Wojska Polskiego 28 St., 60-637 Poznan, Poland*

Author for correspondence e-mail: [pierrot@up.poznan.pl](mailto:pierrot@up.poznan.pl)

Materials and methods

**Experimental Design.** Five combinations of seedlings and mulching with fresh leaves of *P*. *serotina* were established using the seedlings of *Prunus serotina* and *Quercus petraea*.

Fig. S1. The combinations of *Quercus petraea* and *Prunus serotina* seedlings planted in pots and distributed in three blocks and three light treatments: 10, 25, and 100 % of full sun light. The *P*. *serotina* roots from the combinations P and Q + P + L (in rectangle) were used for the prunasin analyses and these combinations were the object of the present study. For biometrical and physiological measurements three and two seedlings per species per pot were used. Q – three *Q*. *petraea* seedlings, P – three *P*. *serotina* seedlings, Q + L - three *Q*. *petraea* seedlings with mulching with *P*. *serotina* leaves, Q + P - three *Q*. *petraea* and six *P*. *serotina* seedlings, Q + P + L - three *Q*. *petraea* and six *P*. *serotina* seedlings with mulching.

**Biomass allocation.** The following equations were used to calculate the different parameters of biomass allocation:

1. Root / Shoot ratio (*R* : *S*, g g^-1^)

$R:S=\frac{W_{R}}{W_{S}+W_{L}}$ (1)

*W_R_* – root dry weigh (g)

*W_S_* – shoot dry weigh (g)

*W_L_* – leaves dry weigh (g)

2. Leaf mass-to-area ratio (*LMA*, g m^-2^)

$LMA=\frac{W_{L}}{A_{L}}$ (2)

*A_L_* – leaf area (m^2^)

3. Leaf weight ratio (*LWR*, g g^-1^)

$LWR=\frac{W_{L}}{W}$ (3)

*W*- total seedling dry weight

4. Leaf area ratio (*LAR*, m^2^ g^-1^)

$$LAR=\frac{A_{L}}{W}$$

**Statistical analyses.** The linear model of ANOVA in split-split plot design was applied to test the effects of sampling time, light and combination of seedlings, and their interactions on prunasin concentration in *P*. *serotina* roots. The model is following:

$$y_{ijkl}=\mu+r_{l}+a_{i}+e_{il}^{(I)}+b_{j}+{(ab)}_{ij}+e_{ijl}^{(II)}+c_{k}+{(ac)}_{ik}+{(bc)}_{jk}+{(abc)}_{ijk}+e_{ijkl}^{(III)}$$

*y_ijkl_* – value of trait measured on plot and obtained at *i* – time of sampling = 1, 2, 3; in *j* – light treatment = 1, 2, 3; in *k* – combination of competition = 1, 2 with or without mulching; in *l* - block = 1, 2, 3

*µ* – mean value of a trait (fixed effect)

*r_l_* – effect of *l* – block (random effect)

*a_i_* – effect of sampling time (fixed effect)

*e_il_*^(I)^ – the first random experimental error (*i*, *l*) – block the first range

*b*_j_ – effect of *j* – light treatment (fixed)

*(ab)_ij_* – effect of two-way interaction between *i* – time of sampling and *j* – light treatment

*e_ijl_*^(II)^ – the second random experimental error (*i*, *j*, *l*) – block of the second range

*c_k_* – effect of k – combination (fixed effect)

*(ac)_ik_* – effect of two-way interaction between *i* – time of sampling and *k* – combination

*(bc)_jk_* – effect of two-way interaction between *j* – light treatment and *k* – combination

*(abc)_ijk_* – effect of three-way interaction between *i* – time of sampling, *j* – light treatment and *k* – combination

*e_ijkl_*^(III)^ – the third random experimental error of (*i*, *j*, *k*, *l*) - plot

Three experimental errors were calculated. *F*-values were calculated for the effect of sampling time using the value of error I (block x sampling date), for the effect of light treatment using error II (block x light treatment + block x sampling time x light treatment) and for the effect of combination and the interaction: sampling time x combination, light x combination and sampling time x light treatment x combination using error III. When ANOVA showed significant differences between the mean values, Tukey’s *a posteriori* test was applied at the significance level α = 0.05.
